# From microbiome to outcome: the cascading effects of combining acid suppressants with anti-tuberculosis therapy

**DOI:** 10.3389/fmed.2026.1959574

**Published:** 2026-09-22

**Authors:** Shiyu Fang, Xiaoman Yang, Fengjun Liu

**Affiliations:** Department of Infectious Diseases, Affiliated Hospital of North Sichuan Medical College, Nanchong, Sichuan, China

**Keywords:** acid suppressants, anti-tuberculosis therapy, gut microbiota, gut-lung axis, tuberculosis

## Abstract

Gastrointestinal adverse reactions occur in up to 71.8% of patients receiving anti-tuberculosis drugs (ATDs), and acid-suppressing agents are frequently co-prescribed to alleviate these symptoms. Direct evidence from human studies demonstrates that ATDs alone reduce gut microbial diversity, deplete short-chain fatty acid (SCFA)-producing bacteria, and promote opportunistic pathogen overgrowth. Concurrently, acid suppressants, particularly proton pump inhibitors (PPIs), raise intragastric pH and facilitate oral-to-gut translocation of bacteria, independently disrupting microbiota composition and metabolic pathways. In this review, we systematically examine the distinct effects of ATDs and acid suppressants on the gut microbiota, the role of the gut-lung axis in tuberculosis immunity, and, on the basis of mechanistic evidence, propose a theoretical cascade under dual exposure: synergistic microbiota depletion, dual metabolic pathway inhibition, immune homeostasis imbalance, and potentially worsened clinical outcomes. This cascade represents a hypothesis-generating framework derived from preclinical and mechanistic studies, rather than an established clinical pathway, and the critical links from dysbiosis to delayed sputum conversion, poor lesion resolution, or increased drug-induced liver injury currently lack direct human confirmation. On the basis of the available evidence, we recommend that clinicians strictly follow indications for acid suppressants, prefer H₂ receptor antagonists(H_2_RAs) over PPIs, and limit treatment to the shortest effective duration. Probiotics and other microbiota-targeted interventions show promise as adjunctive strategies, but their long-term efficacy remains to be validated. Large-scale prospective studies are urgently needed to test the hypothesized cascade, confirm the direct impact of combined therapy on treatment outcomes, and establish microbiota-based biomarkers for predicting therapeutic response.

## 1 Introduction

Tuberculosis remains a major global public health challenge. The latest “Global Tuberculosis Report 2025” published by the World Health Organization indicates that in 2024, there were approximately 10.7 million new tuberculosis cases worldwide, and more than 1.2 million people died from the disease; tuberculosis has once again become the leading cause of death from a single infectious agent globally (1). Although standardized ATD regimens (2HRZE/4HR regimens) can achieve a cure rate of over 90% for non-drug-resistant tuberculosis, gastrointestinal adverse reactions are extremely common during treatment (clinical data show an incidence rate as high as 71.8%) (2), manifesting as nausea, vomiting, loss of appetite, heartburn, abdominal pain, and bloating. In severe cases, these symptoms can affect patient adherence to medication and even lead to treatment discontinuation (3).

To address these gastrointestinal symptoms, clinicians often routinely co-administer acid-suppressing agents to alleviate patient discomfort. However, existing guidelines still have gaps regarding recommended management strategies for adverse reactions during ATD. While some national and international guidelines mention the use of acid-suppressing agents, they lack specific recommendations on drug types and dosages and rarely provide systematic assessments of the potential risks associated with such combination therapy. Of note, both components of this combination therapy can independently disrupt the gut microbiota. Antituberculosis drugs, as potent, broad-spectrum antimicrobial therapies, have been shown to reduce gut microbial diversity and deplete beneficial bacteria, with effects that can persist for several months after the end of treatment (3, 4). Meanwhile, a growing body of research indicates that acid-suppressing agents, particularly proton pump inhibitors (PPIs), reshape the gut microbiota through a distinct mechanism: by irreversibly inhibiting the gastric H⁺/K⁺-ATPase and raising intragastric pH, they weaken the gastric acid barrier and thereby promote the translocation of oral bacteria into the gut. As a result, PPI users show reduced gut microbial diversity, marked enrichment of oral-derived taxa (such as *Streptococcus* and *Rothia*) and opportunistic pathogens of the phylum Proteobacteria, as well as depletion of butyrate-producing genera such as *Roseburia* and *Faecalibacterium* (5–9); these effects are most pronounced with PPIs, whereas H2 receptor antagonists exert comparatively milder disruptions (10).

As the body's largest immune organ, the gut maintains a bidirectional “gut–lung axis” dialogue with the immune status of the lungs through its symbiotic microbial communities, via mechanisms such as metabolic byproducts and immune cell migration. In the specific context of tuberculosis, the homeostasis of the gut microbiota plays a critical regulatory role in the host's anti-tuberculosis immune response, and microbiota dysbiosis can reshape the expression profile of pulmonary inflammatory factors, affect the phagocytic function of alveolar macrophages, and remotely regulate the differentiation and output of myeloid cells in the bone marrow, thereby influencing the effector function of pulmonary T cells, which is the core component of the host's control over *Mycobacterium tuberculosis* (Mtb) infection (11).

Given this background, an important clinical question has gradually come to the fore: since antituberculosis drugs have already caused an imbalance in the gut microbiota, whether the combination with acid-suppressing agents will further exacerbate dysbiosis and, consequently, affect treatment outcomes has become an urgent clinical and scientific issue requiring clarification. In this review, we aim to summarize research progress regarding the effects of ATDs on the gut microbiota, the differential effects of PPIs and other types of acid-suppressing agents on the gut microbiota, and the mechanisms of action of the “gut–lung axis” in tuberculosis. We also explore the potential associations and mechanisms of action within the gut microbiota-immunity-therapeutic efficacy causal chain under the dual exposure scenario of “anti-tuberculosis drugs plus acid-suppressing agents,” with the aim of providing a reference for the design of future clinical studies and the optimization of acid-suppression strategies in ATD.

## 2 Effects of ATD on the gut microbiota

ATD regimens (taking the standard first-line four-drug combination regimen as an example) must be continued for at least 6 months; this prolonged, high-intensity antibiotic exposure exerts a significant and persistent impact on the gut microbiota. Recent studies have revealed the full scope of this impact across multiple dimensions, including changes in microbiota diversity, community structure, and function (4, 12).

### 2.1 Decreased diversity and structural remodeling: characteristics of “microbiota dysbiosis” induced by ATD

Multiple studies consistently show that standard first-line ATD significantly reduces gut microbial diversity (13–15). Systematic reviews and meta-analyses indicate that ATD results in a sustained decline in gut microbiota diversity across both spatial and temporal scales (4, 16), and that the treatment's impact on gut microbial diversity is more pronounced than on pulmonary microbiota (4, 11). Notably, the effects of different drugs are not consistent. Animal studies suggest that rifampin may be the primary driver of microbiota disruption, whereas isoniazid monotherapy may even be associated with an increase in certain beneficial bacterial genera (12, 13). Longitudinal studies further reveal that, at the microbiota composition level, persistent patterns of imbalance are observed during treatment, such as a decrease in the relative abundance of the Bacteroidetes phylum and an increase in the Proteobacteria phylum (4, 16, 17). Furthermore, ATD not only affects bacterial composition but also leads to concurrent changes in the fungal community. A cross-domain network analysis revealed that, after 2 months of ATD, the bacterial-fungal interaction network in patients with active pulmonary tuberculosis became further simplified, with the network exhibiting a simple structure comprising only 23 genera, suggesting that ATD exacerbates the overall disruption of microecological stability (18).

### 2.2 Key microbial changes: depletion of beneficial bacteria and increase in opportunistic pathogens

One of the most prominent ecological consequences of ATD is a significant reduction in SCFA-producing beneficial bacteria (19). SCFAs (primarily including acetic acid, propionic acid, and butyric acid) are not only an important energy source for intestinal epithelial cells but also participate in host immune regulation, maintenance of intestinal barrier integrity, and suppression of inflammatory responses by activating G protein-coupled receptors and inhibiting histone deacetylases (20). Evidence shows that standard ATD can lead to a decline in key SCFA-producing bacteria such as the genus *Ruminococcus* and the genus *Faecalibacterium*, and these changes are associated with immune dysregulation and a potential reduction in treatment efficacy (21). *Roseburia* is one of the primary producers of butyrate; a decrease in its abundance can directly reduce butyrate levels in the intestinal lumen, thereby impairing the differentiation and function of regulatory T cells. *Faecalibacterium* (particularly *Faecalibacterium* prausnitzii) is known for its anti-inflammatory properties, and its reduction is closely associated with increased intestinal permeability and elevated systemic inflammation levels (22, 23).

At the genus or species level, multiple studies have observed a more widespread depletion of commensal bacteria, with anaerobic commensals such as *Clostridium*, *Ruminococcus*, *Faecalibacterium*, *Lactobacillus*, and *Bifidobacterium* all showing a decreasing trend (4, 14, 19). These genera play an irreplaceable role in maintaining the homeostasis of the gut microbiota: certain members of the *Clostridium* genus (such as *Clostridium* clusters IV and XIVa) are major contributors to butyrate production, and their reduction directly impairs the gut's energy metabolism and immunoregulatory functions (24). *Treponema* spp. are involved in cellulose degradation and SCFA production; a decline in their abundance can further exacerbate SCFA deficiency. *Bifidobacterium* and *Lactobacillus*, as classic representatives of probiotics, play key roles in competitively excluding pathogens, maintaining the intestinal barrier, and regulating mucosal immunity.

Corresponding to the reduction in beneficial bacteria, the relative abundance of certain conditionally pathogenic bacteria has increased; among them, *Bacteroides* (such as *Bacteroides fragilis*) and some opportunistic pathogens (such as *Enterococcus*) show elevated relative abundances during treatment (25). The overgrowth of *Bacteroides fragilis* warrants particular attention, although it is a component of the normal intestinal microbiota, enterotoxigenic strains (ETBF) carry enterotoxin genes and, against a backdrop of microbial imbalance, may breach the mucosal barrier and contribute to inflammatory responses and systemic infections. The genus *Enterococcus* (particularly *Enterococcus faecalis* and *Enterococcus* faecium) is a major pathogen responsible for nosocomial infections; its proliferation not only increases the risk of secondary infections but, due to its inherent and acquired drug resistance, may also serve as a potential reservoir for the transmission of drug-resistance genes within the gut microbiota against the backdrop of microbial imbalance.

In summary, standard ATD inflicts a significant and persistent disruption on the gut microbiota through its broad-spectrum antimicrobial activity. Its core characteristics are manifested as: a sustained decline in *α*-diversity; significant depletion of beneficial bacterial genera that produce SCFAs (such as *Ruminococcus* and *Faecalibacterium*); relative proliferation of opportunistic pathogens (such as *Bacteroides fragilis* and *Enterococcus*); and concurrent simplification of the fungal community. This microbial imbalance not only weakens the intestinal barrier and metabolic functions but also lays the groundwork for microbial vulnerability to the subsequent “dual hit” effect when combined with acid-suppressing agents.

## 3 Effects of acid-suppressing agents on the gut microbiota

The above evidence indicates that ATD itself imposes significant and persistent disruption on the gut microbiota. However, in clinical practice, a considerable proportion of tuberculosis patients experience marked gastrointestinal adverse reactions after initiating ATD and consequently use acid-suppressing agents, primarily PPIs, in combination; these drugs themselves also have a significant impact on the gut microbiota that cannot be ignored.

### 3.1 Core mechanisms by which acid-suppressing agents affect the gut microbiota

Stomach acid serves as the body's first line of defense against the entry of oral microorganisms into the lower gastrointestinal tract. Acid-suppressing agents, such as PPIs, irreversibly block the H⁺/K⁺-ATPase in gastric parietal cells, thereby potently inhibiting gastric acid secretion and causing a significant rise in intragastric pH (5). This change weakens the bactericidal effect of gastric acid on orally ingested microorganisms, allowing bacteria of oral origin (which would normally struggle to survive in an acidic environment) to survive their passage through the gastrointestinal tract and ultimately colonize the gut. This process is known as “oral-to-gut translocation.” In recent years, multiple studies with rigorous experimental designs have validated this mechanism. For example, Xiao et al. analyzed changes in oral and gut microbiota before and after intervention via metagenomic sequencing 7 days after treatment with acid-suppressing agents. The results suggested that oral bacteria had migrated to the gut, leading to a significant increase in the number and total abundance of oral-origin species in the gut. This prospective intervention study further elucidated the mechanism: after 7 days of PPI administration, the abundance of *Streptococcus anginosus* in the gut microbiota increased 42-fold compared to baseline (6). Additionally, an *in vitro* experiment confirmed that *Streptococcus anginosus* cannot survive in an environment with a pH below 5, providing direct experimental evidence for the gastric acid barrier hypothesis (6).

In addition to indirectly regulating the gut microbiota by affecting gastric pH, some recent studies suggest that PPIs may also contribute to the remodeling of the gut microbiota through direct antibacterial effects; however, the significance of this effect remains controversial and may be species-selective (26–28). *In vitro* single-culture experiments show that omeprazole does not significantly inhibit 21 common gut commensal bacteria within the physiological concentration range; it exerts only a slight effect on some members of the *Bacteroides* order at supraphysiological concentrations, suggesting that under routine dosing conditions, PPIs have limited direct inhibitory effects on the growth of most commensal bacteria (27). However, some studies have observed that PPIs exert direct inhibitory effects on specific microorganisms. *In vitro* experiments indicate that pantoprazole and esomeprazole can significantly inhibit the proliferation of the intestinal protozoan *Blastocystis* and *Candida albicans*, with their efficacy even surpassing that of metronidazole at high concentrations (26); the direct antibacterial activity of PPIs against *Helicobacter pylori* (H. pylori) has also been confirmed. These findings suggest that the direct antibacterial effects of PPIs are primarily limited to specific microbial groups (such as fungi, protozoa, and *Helicobacter pylori*) (26, 29). Based on the available evidence, the impact of PPIs on the gut microbiota may be characterized as “primarily indirect (pH-mediated oral-to-gut translocation) and secondarily direct (antibacterial/bactericidal effects on specific bacterial species).”

Finally, the mechanisms described above should not be assumed to operate on an intact ecosystem. In patients receiving ATD, the gut community has already been depleted of anaerobic, SCFA-producing commensals (4, 19), and the attendant decline in butyrate availability weakens epithelial barrier integrity as well as colonization resistance—the capacity of a resident community to restrict the establishment of incoming organisms (30). Because PPI-mediated oral-to-gut translocation delivers a continuous influx of oral-origin taxa precisely under these conditions, the incoming organisms meet reduced competition and a larger pool of unoccupied niches than they would in an undisturbed gut, and may therefore be more likely to persist than to pass through transiently. Viewed mechanistically, the two exposures act on the same ecosystem sequentially rather than in parallel: ATD lowers the community's resistance to perturbation, while acid-suppressing agents provide the perturbation. This inference, and its consequences for community composition and anti-tuberculosis immunity, are taken up in Sections 4, 5.

### 3.2 Changes in microbiota composition

The use of acid-suppressing agents is closely associated with a reduction in gut microbial diversity (5). Large-scale population studies have shown that the number of observed species and the Shannon index are significantly lower in users of acid-suppressing agents, suggesting that both the richness and evenness of their gut microbiota are adversely affected. However, the diversity signal is not uniform across designs. It rests on large cross-sectional cohorts vulnerable to confounding by indication: Imhann et al. (211 users vs. 1,604 non-users across three cohorts, *n* = 1,815) reported significantly reduced *α*-diversity and changes in 20% of taxa (5), and Jackson et al. (1,827 twins) likewise found reduced *α*-diversity with enrichment of oral commensals (7). The prospective within-subject study of Reveles et al., however, found a different pattern: in 24 healthy older adults given omeprazole 20 mg daily for 14 days, neither OTU richness (485 ± 84.3 vs. 496 ± 88.7, *p* = 0.32) nor the Shannon index (3.86 ± 0.27 vs. 3.92 ± 0.31, *p* = 0.28) changed, whereas *β*-diversity was markedly altered (PERMANOVA *p* < 0.0001) (31). Crucially, an unchanged *α*-diversity does not imply an absence of effect: over the same 14 days *Streptococcaceae* increased roughly fourfold (1.49% to 5.93%, *p* = 0.0009) while butyrate-producing *Lachnospiraceae* (*p* = 0.0059) and *Bifidobacteriaceae* (*p* = 0.0275) declined significantly (31). The findings are thus best reconciled as a temporal sequence—composition is reshaped first, and richness falls only with sustained exposure (9).

At the microbial community structure level, large-scale studies have shown that PPI users exhibit a decrease in the relative abundance of the Bacteroidetes phylum in the gut, while the Proteobacteria phylum (which includes various opportunistic pathogens) is enriched (5). The most prominent change at the genus level is the significant expansion of oral-derived microorganisms (such as *Streptococcus* and *Rothia*), which directly corroborates the “oral-to-gut translocation” mechanism (8). One notable difference is that, unlike antituberculosis drugs, which generally suppress the genera *Bifidobacterium* and *Lactobacillus*, some prospective studies have found that the relative abundance of these two genera actually increases after short-term PPI treatment (8). This may be due to elevated gastric pH, which makes it easier for these bacteria to survive and reach the intestine (32). However, consistent with the effects of antituberculosis drugs, PPIs also lead to a decrease in key butyrate-producing bacterial genera (*Roseburia* and *Faecalibacterium*) (4, 19). This “additive” effect on key beneficial bacteria suggests that the impact on intestinal immune homeostasis when ATDs are used in combination with acid-suppressing agents may not be simply additive.

### 3.3 Functional remodeling and metabolic pathways

The remodeling of the gut microbiota's functional profile due to PPI exposure should also not be overlooked. First, the impairment of SCFA synthesis pathways has been repeatedly confirmed in PPI users. Fecal metabolomics analysis shows that levels of butyrate and total SCFAs in the feces of long-term PPI users are significantly lower than those of non-users, consistent with the decline in the abundance of SCFA-producing bacterial genera (such as *Ruminococcus* and *Faecalibacterium*) (33). A deficiency in butyrate not only directly affects the energy supply to intestinal epithelial cells and the integrity of the intestinal barrier but may also influence the establishment of mucosal immune tolerance by reducing the differentiation of local intestinal Tregs. Furthermore, evidence from animal studies has further expanded our understanding at the functional level; some studies have found that long-term exposure to rabeprazole can induce gut microbiota dysbiosis in mice and reduce bile acids in the ileum, particularly 7-ketolithocholic acid (7-KLCA) and chenodeoxycholic acid (CDCA) (34). Bile acids are emulsifiers essential for the digestion and absorption of lipids; they also act as key signaling molecules that participate in the regulation of host energy metabolism, inflammatory responses, and the innate immune response of the gut by activating the farnesoid X receptor (FXR) and the G protein-coupled bile acid receptor (TGR5) (34–36). The reduction in 7-KLCA may affect bile acid metabolic homeostasis and intestinal mucosal repair by lifting the inhibition of the intestinal FXR-FGF15 axis (Table 1).

**Table 1:** Comparison of the effects of anti-tuberculosis drugs and acid suppressants on the gut microbiota.

Parameter	ATD	Acid suppressants (PPI-predominant)	Dual exposure: synergistic effects
Alpha diversity (Shannon index, etc.)	↓↓ (persists for months after treatment)	↓ (long-term) ↔ (short-term)	↓↓↓ (synergistic; delayed recovery)
SCFA-producing bacteria (Roseburia, Faecalibacterium, etc.)	↓↓ (dominant depletion)	↓↓ (direct suppression)	↓↓↓ (synergistic aggravation)
Opportunistic pathogens (B. fragilis, Enterococcus, etc.)	↑ (ETBF expansion)	↑ (Proteobacteria enriched)	↑↑ (additive; increased risk of infection)
Oral-derived microorganisms (Streptococcus, Rothia, etc.)	↔ (no direct effect)	↑↑ (oral-to-gut translocation hallmark)	↑↑ (42-fold increase)
Probiotic groups	↓↓ (broad-spectrum inhibition)	↑ (enhanced survival due to a reduced acid barrier)	↔ (Directionally antagonistic)
Butyrate/total SCFA production	↓↓	↓↓	↓↓↓ (synergistic exhaustion)
Secondary bile acid transformation	↓↓ (loss of substrate-producing bacteria)	↓ (direct impact on the bile acid pool)	↓↓ (dual blockade)
LPS translocation burden	↑ (barrier disruption)	↑ (barrier disruption)	↑↑ (positive feedback acceleration)

ATD, anti-tuberculosis drugs; PPI, proton pump inhibitor; SCFAs, short-chain fatty acids; ETBF, enterotoxigenic *Bacteroides fragilis*; LPS, lipopolysaccharide. Arrows indicate the direction and magnitude of change: ↑/↓ = increase/decrease; ↑↑/↓↓ = marked increase/decrease; ↑↑↑/↓↓↓ = synergistic; ↔ = minimal or no significant change. Synergistic effects under dual exposure are theoretical inferences based on Section 4 of the text and require prospective validation.

### 3.4 Differences among types of acid-suppressing agents and duration of use

The effects of different types of acid-suppressing agents and their duration of use on the gut microbiota vary, and this distinction warrants separate attention. Existing evidence suggests that H_2_ receptor antagonists exert a relatively mild inhibitory effect on gastric acid secretion; their disruptive effects on the gut microbiota are generally weaker than those of PPIs, but long-term use can still cause a significant shift in microbial *β*-diversity, although the magnitude of this shift is typically lower than that observed with PPIs (10, 37). Regarding duration of exposure, large-scale cohort studies show that the longer PPIs are used, the more pronounced the shift in gut microbiota structure becomes, showing a duration-dependent pattern (10); in contrast, short-term treatment (2–4 weeks) typically causes only minor changes (38). Some studies suggest that PPI-related changes in the microbiota may gradually reverse after discontinuation of the medication; however, the rate of recovery and whether the microbiota can fully return to baseline levels remain influenced by multiple factors, including individual differences, baseline microbiota status, and duration of use (9, 39, 40). This characteristic carries particularly significant clinical implications for patients requiring long-term (at least 6 months) ATD therapy.

In summary, the effect of acid-suppressing agents on the gut microbiota is not a unidirectional “change in diversity,” but rather a complex remodeling process that includes the expansion of specific oral-derived taxa and the inhibition of key metabolic pathways (SCFA synthesis and secondary bile acid metabolism). When this remodeling is superimposed on the microbiota disruption already caused by antituberculosis drugs, the stability of the gut microbiota faces a “dual hit” from two fronts (the synergistic depletion of beneficial metabolites and the concurrent expansion of potentially harmful bacterial populations). Whether this “dual hit” at the microbiota level affects the host's antituberculosis immune response via the gut–lung axis will be the central issue explored in the subsequent sections of this paper.

## 4 Mechanisms of action of the gut–lung axis in tuberculosis immunity

### 4.1 The concept of the gut–lung axis and bidirectional signaling pathways

The gut–lung axis refers to a bidirectional regulatory network connecting the gut and lungs via microbial communities, metabolites, and immune signaling molecules; it serves as a critical hub in systemic immunity (41, 42). This concept has evolved from an early theoretical hypothesis into a biological framework supported by multi-omics studies and germ-free animal experiments. Current evidence indicates that the gut microbiota not only determines local intestinal immune homeostasis but also significantly influences pulmonary susceptibility to respiratory pathogens (particularly Mtb), the course of infection, and treatment response through distal regulatory mechanisms (41). Signal transduction via the gut–lung axis primarily occurs through the following three pathways: (1) Circulatory transport of microbial metabolites. Short-chain fatty acids (SCFAs), primarily acetic acid, propionic acid, and butyric acid, produced by the gut microbiota through the fermentation of dietary fiber, as well as bioactive molecules such as tryptophan metabolites (e.g., indole-3-acetic acid, indole-3-propionic acid), secondary bile acids, and polyamines, can reach the lungs via the bloodstream. These metabolites exert immunomodulatory functions in distant organs, suppressing pulmonary inflammation and enhancing immune homeostasis. (2) Systemic signaling by microbial structural ligands. Components of the bacterial cell wall [such as lipopolysaccharide (LPS), peptidoglycan, and other microbial-associated molecular patterns (MAMPs)] can enter the circulatory system, where they are recognized by pattern recognition receptors in the lungs, triggering a local innate immune response. (3) Cross-organ migration of immune cells. Gut-derived immune cells [such as activated T cells and dendritic cells (DCs)] can migrate to the lungs via the lymphatic and blood circulatory systems, carrying the immunological imprint of the gut microenvironment and exerting effector functions in the lungs (42–48) (Figure 1).

**Figure 1:**
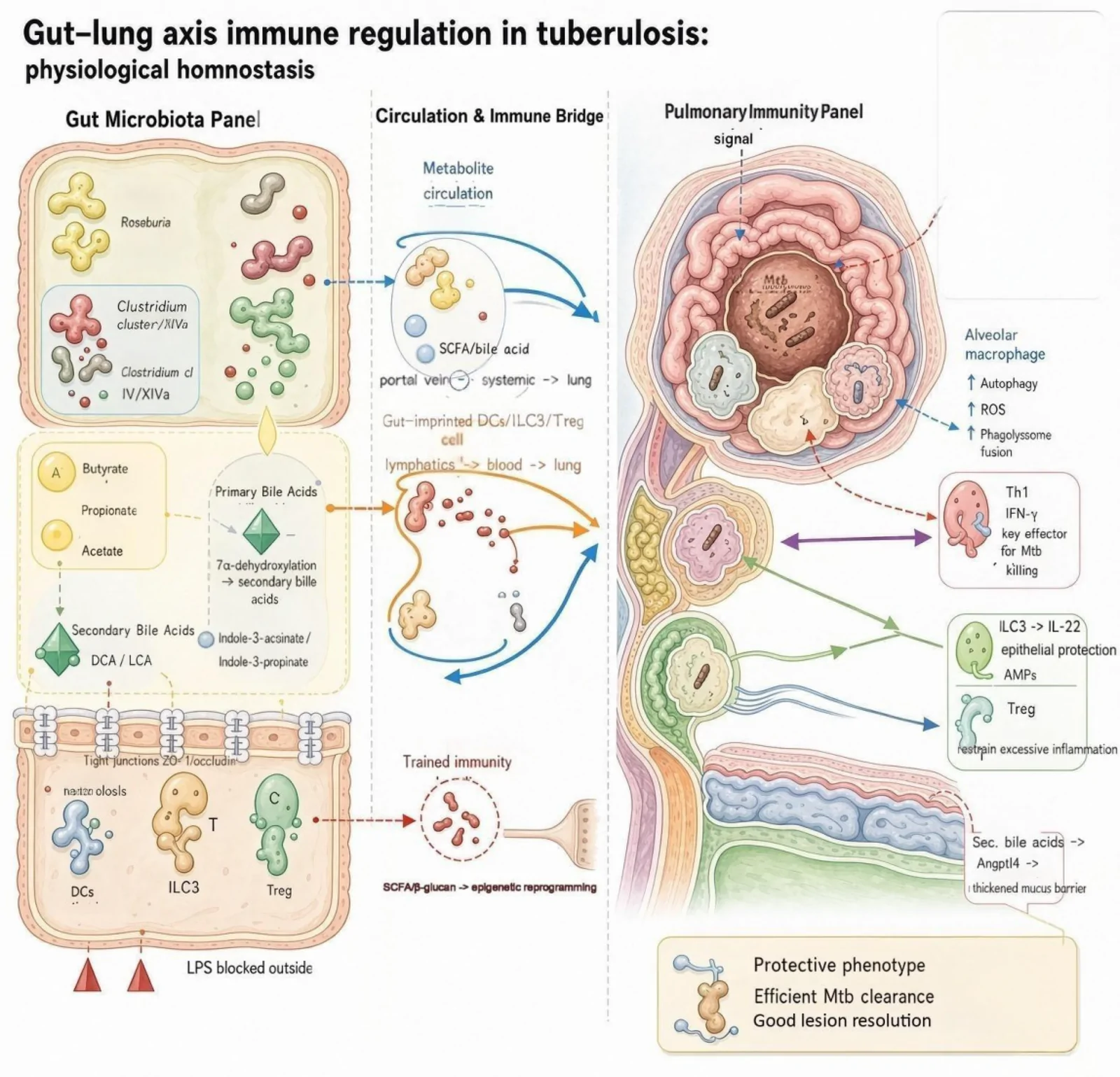
Schematic of three major gut–lung signaling pathways. (1) Metabolites (SCFAs, tryptophan derivatives, bile acids, polyamines) enter the bloodstream and promote pulmonary immune homeostasis. (2) Microbial ligands such as LPS and peptidoglycan reach the lung via circulation, triggering innate immunity via pattern recognition receptors (PRRs). (3) Gut-primed dendritic cells and T cells migrate to the lung to exert tissue-specific effector functions.

### 4.2 The SCFA pathway: from metabolites to immune regulation

SCFAs are the most extensively studied signaling molecules in the gut–lung axis. They are primarily produced by the anaerobic fermentation of dietary fiber by the gut microbiota and exert multifaceted immunoregulatory effects via the circulatory system. At the level of immune cell regulation, SCFAs promote Treg differentiation and finely regulate the Th1/Th17 balance by activating G protein-coupled receptors (such as GPR43/FFAR2, GPR41/FFAR3, GPR109A) and by inhibiting histone deacetylases (HDACs) (49). Th1-derived interferon-*γ* (IFN-*γ*) is a key effector for clearing Mtb and maintaining granuloma structure (50). SCFAs can also reach the lungs via the portal vein and systemic circulation, where they modulate alveolar macrophage function via the same receptors and HDAC inhibition; they enhance phagocytic capacity, mitochondrial respiratory efficiency, and autophagy levels to promote Mtb clearance while upregulating anti-inflammatory mediators such as interleukin-10 (IL-10) and downregulating pro-inflammatory responses, including tumor necrosis factor-α (TNF-α), IL-1β, and IL-6. At the tissue barrier level, as a key energy substrate for the intestinal epithelium, butyrate can upregulate the expression of occludin, claudin, and zonula occludens-1 (ZO-1), promote the secretion of mucins and antimicrobial peptides (such as RegIII*γ*), and inhibit epithelial apoptosis, thereby strengthening the intestinal mechanical and immune barriers (51–56).

### 4.3 Dysbiosis and immune shift: LPS translocation and immune remodeling

However, the impact of gut dysbiosis on the immune response to tuberculosis extends far beyond the depletion of beneficial metabolites such as SCFAs; its core mechanism lies in the “qualitative change” in immune recognition signals triggered by alterations in the microbial community structure. Dysbiosis is often accompanied by disruption of the intestinal epithelial barrier, leading to massive translocation of LPS from Gram-negative bacteria into the bloodstream; circulating LPS induces chronic, low-grade systemic inflammation by continuously activating the Toll-like receptor 4 (TLR4)/nuclear factor-*κ*B (NF-*κ*B) signaling pathway, forcing dendritic cells into a state of non-physiological hyperactivation. This manifests as dysregulation of costimulatory molecule expression and upregulation of IL-10 secretion, severely impairing their ability to present Mtb antigens and drive Th1 polarization (57–60). At the same time, alterations in the gut microbiota's metabolic profile, particularly the inhibition of tryptophan and primary bile acid metabolic pathways, disrupt the normal epigenetic programming of bone marrow hematopoietic stem cells. As a result, the bone marrow no longer produces myeloid cells with “trained immunity” characteristics; instead, it promotes the generation of large numbers of immature or pro-inflammatory myeloid cells. These cells, upon migration to the lungs, exhibit poor bactericidal efficacy and are prone to secreting excessive inflammatory cytokines, which exacerbate pulmonary histopathological damage while failing to control intracellular Mtb replication (61–63). Furthermore, regulation of the gut–lung axis is expanding from “metabolite-mediated” to “cell migration”-mediated mechanisms; alterations in specific gut microbiota composition can drive pathological homing of ILC2s from gut-associated lymphoid tissues to the lungs, where they mediate a Type 2 immune shift by secreting IL-4, IL-5, IL-13. This dysbiosis-induced expansion of ILC2 not only directly antagonizes the protective immunity dominated by Th1/ILC1 cells but also, by inducing M2 macrophage polarization, creates an immunosuppressive microenvironment in the lungs that favors Mtb latency or replication, thereby establishing a framework of pathological immune remodeling centered on abnormal DC activation, myeloid differentiation bias, and ILC2 dysregulation (64–66).

### 4.4 Secondary bile acid metabolic pathways: FXR/TGR5 signaling and antimicrobial defense

As another key hub in gut microbiota metabolism, secondary bile acids play a unique role, complementary to that of SCFAs, in immune regulation along the gut–lung axis via FXR- and TGR5-mediated signaling pathways. Their production is highly dependent on the 7*α*-dehydroxylation of primary bile acids by strictly anaerobic bacteria, such as the *Clostridium* cluster; therefore, they are highly susceptible to depletion in the context of ATD and dysbiosis (67). At physiological concentrations, secondary bile acids, such as deoxycholic acid (DCA) and lithocholic acid (LCA), precisely regulate the expression of antimicrobial peptides and ANGPTL4, thereby maintaining intestinal barrier integrity. They also enhance the intracellular bactericidal activity of alveolar macrophages and monocytes via the TGR5-cyclic adenosine monophosphate (cAMP) signaling axis, including by promoting autophagosome-lysosome fusion and reactive oxygen species (ROS) production, thereby directly limiting the intracellular survival of Mtb (68, 69). At the same time, secondary bile acids serve as a crucial bridge linking innate lymphoid cells (ILCs) to the adaptive immune system (70). Through FXR, they regulate the secretion of interleukin-22 (IL-22) by intestinal type 3 innate lymphoid cells (ILC3); IL-22 not only strengthens epithelial barrier function but also acts on the lungs via the circulation, promoting the host's early defense against intracellular pathogens. However, dysbiosis leads to the depletion of secondary bile acid-producing bacteria and the suppression of FXR/TGR5 signaling, which not only directly weakens the aforementioned antibacterial and bactericidal effects but also triggers a significant immune shift: The absence of FXR agonists leads to impaired ILC3 function and reduced IL-22 secretion, weakening early defense mechanisms while simultaneously lifting the restraint on Th17 cell hyperactivation, resulting in abnormally elevated IL-17A levels and exacerbating inflammatory pathological damage in the lungs (71, 72). Furthermore, secondary bile acid deficiency disrupts the metabolic reprogramming of macrophages, hindering their transition from glycolysis to oxidative phosphorylation, which results in energy metabolism disorders and impaired bactericidal capacity (73). This “antimicrobial peptide deficiency, ILC3/Th17 imbalance, macrophage metabolic dysfunction” triad, triggered by secondary bile acid depletion, combines with the aforementioned SCFA deficiency and LPS translocation effects to collectively constitute the underlying metabolic and immunological mechanisms of gut–lung axis dysfunction in the context of dysbiosis, profoundly revealing the decisive role of host-microbiota co-metabolites in maintaining anti-tuberculosis immune homeostasis.

## 5 The causal chain of microbiota–immunity–therapeutic efficacy under dual exposure to “antituberculosis drugs+acid-suppressing agents”

The combined use of antituberculosis drugs (ATDs) and acid-suppressing agents constitutes a “dual hit” to the gut microbiota and systemic immune function, and its disruptive effects on the microbiota may far exceed the sum of the effects of either factor alone. Based on the microbiota changes described earlier, this section will explore how these two interventions, through synergistic effects, amplify microbiota dysbiosis into immune dysfunction and ultimately influence clinical treatment outcomes (Table 2).

**Table 2:** Dual-mode regulation of gut–lung axis immunity: physiological homeostasis versus pathological shift under dual exposure.

Signaling axis (metabolite → receptor)	Physiological homeostasis (normal microbiota)	Pathological shift (after dual exposure)	Key immunological outcome node
SCFAs → GPR41/43	Activates alveolar macrophage autophagy and respiratory burst; maintains the Th1/Treg balance	Signal attenuation → impaired macrophage killing; weakened Th1 responses	↓ intracellular Mtb clearance+↓ granuloma stability
SCFAs → HDAC inhibition	Promotes Treg differentiation and suppresses excessive inflammation	De-repression → aberrant Th17 activation, ↑ IL-17A	Aggravated pulmonary immunopathology (expanded necrosis)
Secondary bile acids (DCA/LCA) → FXR	Induces ANGPTL4 and antimicrobial peptides; upregulates ILC3-IL-22 for barrier repair	Loss of agonism → ↓ antimicrobial peptides; ↓ IL-22	Simultaneous disruption of gut–lung barriers; enhanced microbial translocation
Secondary bile acids (DCA/LCA) → TGR5	Enhances bactericidal activity of monocytes and macrophages (↑ ROS)	Signal disruption → failure of metabolic reprogramming (transition from glycolysis to OXPHOS blocked)	Energy deficit → inability to support killing functions
LPS → TLR4/NF-*κ*B (bypass signal)	Mostly sequestered by the gut barrier; low-level signaling	Massive translocation → chronic activation	DC overactivation, impaired antigen presentation, ↑ IL-10 (suppresses Th1 polarization)
Pathological homing signal (novel pathway)	ILC2 reside in the gut, maintaining homeostasis	Microbiota-driven pathological homing of ILC2 to the lungs	Type-2 immune deviation (↑ IL-4/IL-5), M2 macrophage polarization → creates a permissive niche for Mtb

SCFA, short-chain fatty acid; GPR, G-protein-coupled receptor; HDAC, histone deacetylase; Treg, regulatory T cell; Th, T helper cell; IL-22, interleukin-22; ILC, innate lymphoid cell; ROS, reactive oxygen species; LPS, lipopolysaccharide; TLR4, Toll-like receptor 4; NF-κB, nuclear factor kappa-B; DC, dendritic cell; Mtb, *Mycobacterium tuberculosis.*

### 5.1 Synergistic microbial depletion and intestinal barrier disruption

At the level of microbiota disruption, the synergistic effect of ATDs and acid-suppressing agents far exceeds simple addition. ATDs directly inhibit the colonization of SCFA-producing bacteria from within, while PPIs introduce non-commensal bacteria (such as *Streptococcus* species) from the outside via oral-to-gut translocation, allowing them to occupy ecological niches; together, they form a “dual attack from within and without.” This combined effect results in a greater loss of key bacterial genera (such as *Roseburia* and *Faecalibacterium*) and a more pronounced decline in butyrate levels than either factor alone could cause, leading to deeper damage to SCFA-mediated immune regulatory pathways and creating a vicious cycle with intestinal barrier disruption.

### 5.2 Dual blockage of metabolic pathways

At the metabolic level, ATD-induced depletion of the *Clostridium* cluster and PPI-induced reduction of the ileal bile acid pool create a dual blockage. The former limits the conversion of primary to secondary bile acids (DCA, LCA), while the latter reduces key molecules such as 7-KLCA and CDCA; the combination of these two factors results in a more profound inhibition of the FXR/TGR5 signaling pathway than either factor alone. This profound blockage of the metabolic pathway not only weakens the expression of antimicrobial peptides and the function of the intestinal immune barrier, as described earlier, but may also indirectly affect systemic anti-infective defenses through FXR-mediated metabolic-immune crosstalk.

### 5.3 Immune homeostasis dysregulation and systemic inflammation

At the level of immune dysregulation, dual exposure exacerbates systemic inflammation and immune homeostasis imbalance. The increased intestinal permeability induced by the combined use of ATD and acid-suppressing agents allows for the massive translocation of microbiota-associated molecular patterns, such as LPS, into the bloodstream, inducing chronic low-grade inflammation via the TLR4/NF-*κ*B pathway; simultaneously, alterations in gut microbiota composition and metabolites may disrupt the balance of the pulmonary immune microenvironment, resulting in a relative weakening of the protective Th1 response and abnormal activation of Th17-related pathways. This immune imbalance may compromise the structural stability of granulomas, creating conditions for the persistence or reactivation of Mtb (74).

### 5.4 Risk of drug-induced liver injury

In terms of clinical outcomes, the effects of ATDs and acid-suppressing agents are primarily reflected in two aspects: reduced hepatic tolerance and delayed treatment response. Drug-induced liver injury (DILI) is one of the leading causes of ATD discontinuation and treatment failure, with an incidence of 9.5%–14.1% for anti-tuberculosis drug-induced liver injury (ATB-DILI) in China (75); the dual exposure to ATDs and acid-suppressing agents may increase susceptibility to DILI through multiple mechanisms. First, regarding bile acid metabolism, the gut microbiota dysbiosis jointly caused by ATDs and acid-suppressing agents further reduces the production of secondary bile acids and weakens FXR activation. This not only disrupts the homeostasis of intrahepatic bile acid synthesis and efflux but also lowers the intrinsic resistance of hepatocytes to drug toxicity. Second, regarding drug metabolism, as a key regulator of hepatic xenobiotic metabolism, the gut microbiota can, through structural alterations, modify the expression and activity of the cytochrome P450 (CYP450) enzyme system, leading to abnormal metabolic rates of drugs such as isoniazid and rifampin (76, 77). This affects the balance between the formation and clearance of toxic intermediates (such as acetylhydrazine and hydrazine in isoniazid), thereby altering susceptibility to liver injury. PPIs themselves can also interact pharmacokinetically with antituberculosis drugs by inhibiting metabolic enzymes such as CYP2C19, further complicating the risk of liver damage (78). Meanwhile, at the inflammatory and immune levels, LPS translocation caused by damage to the intestinal epithelial barrier enters the liver via the portal vein, activating Kupffer cells and triggering a storm of pro-inflammatory factors such as TNF-α and IL-1β (79, 80). These factors not only directly damage hepatocytes but also produce synergistic toxic effects with antituberculosis drugs. However, the aforementioned mechanisms are primarily based on animal studies and theoretical inferences; direct clinical evidence regarding the risk of DILI associated with the combination of ATDs and acid-suppressing agents remains insufficient and requires validation through prospective studies.

### 5.5 Delayed treatment response and lesion resolution

Another significant impact of the dysregulated microbiota-immune axis on treatment outcomes lies in the weakening of pulmonary immune surveillance, which in turn leads to slow lesion resolution and impaired bacterial clearance. Alveolar macrophages, as the core effector cells responsible for clearing Mtb, may suffer severely impaired bactericidal capacity under conditions of dual exposure: depletion of SCFAs and secondary bile acids could impair macrophage autophagy induction and respiratory burst capacity via the GPR41/GPR43 and FXR/TGR5 signaling pathways and disrupt the metabolic adaptive reprogramming that macrophages undergo during Mtb infection, thereby limiting the energy and substrates required for bactericidal activity. Functionally impaired macrophages would not only struggle to clear intracellular Mtb but might also serve as intracellular “sanctuaries” that allow the bacteria to persist (81). By analogy, dysbiosis-induced immune skew could disrupt the structural integrity of granulomas (Th1/ILC1-mediated protective immune responses may be suppressed, accompanied by abnormal activation of Th17-related pathways and a relative imbalance in the Treg response), potentially leading to a loose granuloma structure and an expanded central necrotic area that hinders the effective infiltration of effector immune cells into the lesion core and creates conditions for Mtb to persist in the hypoxic, nutrient-deprived center of the lesion. It should be emphasized that most of the mechanistic evidence described above is extrapolated from studies of other pathogens, *in vitro* experiments, and animal models, whereas direct evidence in patients with tuberculosis is currently limited to the well-characterized dysbiosis induced by anti-tuberculosis therapy itself and to observational associations between gut dysbiosis and TB treatment response (82, 83). Consequently, although the aforementioned decline in immune function could plausibly translate into delayed treatment responses (manifesting as delayed cavitary healing on imaging, incomplete lesion resolution, and prolonged time to sputum culture conversion), but these links remain hypothetical, and no prospective clinical study to date has directly demonstrated that acid-suppressant-associated gut dysbiosis delays sputum culture conversion or worsens treatment outcomes in patients with TB. Furthermore, the coexistence of systemic inflammation and a locally immunosuppressive microenvironment may, in theory, create favorable conditions for systemic dissemination of Mtb, thereby increasing the risk of extrapulmonary tuberculosis.

## 6 Discussion

### 6.1 Mechanistic evidence

Integrating the evidence presented in Sections 1–4, a coherent cascade emerges through which dual exposure to ATDs and acid-suppressing agents may impair tuberculosis treatment outcomes (Figure 2). First, ATDs and acid-suppressing agents, particularly PPIs, each independently disrupt gut microbial composition, depleting SCFA-producing genera and expanding opportunistic pathobionts, with some changes persisting for weeks after drug discontinuation (3, 5, 7, 82). Second, these compositional shifts translate into functional alterations, including reduced SCFA production, altered secondary bile acid metabolism, and increased intestinal permeability with LPS translocation (47, 48, 69). Third, through the gut–lung and gut–liver axes, these perturbations converge on pulmonary immune surveillance (impairing alveolar macrophage function, neutrophil homeostasis, and regulatory T-cell responses) and on hepatic inflammatory pathways (46, 52, 76, 83). Finally, when the two drug classes are combined, their effects on microbiota depletion, metabolic pathway blockade, and immune dysregulation are at least additive and potentially synergistic, constituting the “dual hit” cascade proposed in this review. Notably, although each individual link is supported by mechanistic or preclinical data, the full chain, from dual drug exposure to adverse clinical outcomes, remains to be validated in prospective human studies, a point elaborated in Section 5.3. These four steps correspond to the four tiers of the waterfall model illustrated in Figure 2, which proceeds from upstream drug exposure through microbiota disruption and immune-metabolic reprogramming to downstream clinical consequences.

**Figure 2:**
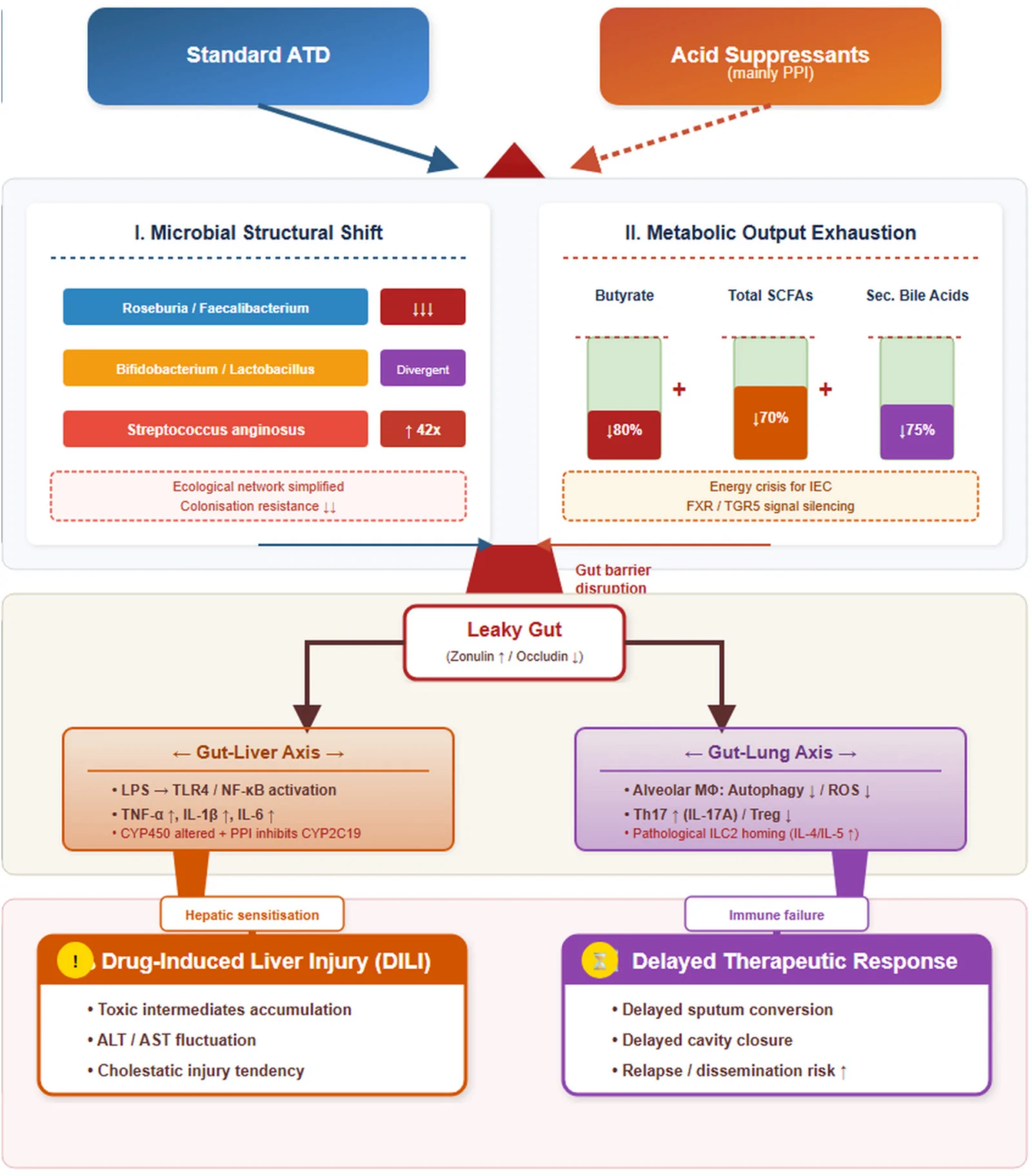
A hypothetical model of the “dual hit” cascade linking combined exposure to antituberculosis drugs (ATDs) and proton pump inhibitors (PPIs) to potentially impaired tuberculosis treatment outcomes. The four tiers of the cascade, proceeding from upstream drug exposure to downstream clinical consequences, are described in detail in Section 5. DILI, drug-induced liver injury.

### 6.2 Clinical implications

#### 6.2.1 Rational use of acid-suppressing agents in ATD

Current TB treatment guidelines provide little guidance on managing gastrointestinal adverse reactions during ATD. The WHO consolidated tuberculosis guidelines (April 2025) give no specific recommendations on the indications, choice, dosage, or duration of acid-suppressing agents (84), and the 2025 guidelines of the American Thoracic Society (ATS), the U.S. Centers for Disease Control and Prevention (CDC), and the European Respiratory Society (ERS) similarly do not address this issue (85). Given this gap and the “dual hit” mechanism described above, we propose the following principles for the use of acid-suppressing agents in ATD:

Strictly adhere to indications. Many ATD-associated symptoms (nausea, heartburn, abdominal pain) reflect direct gastrointestinal irritation or dysbiosis-related reactions rather than acid-related disease. For mild symptoms, prefer adjusting the dosing regimen (dividing doses, taking medication after meals) or short-term symptomatic management over routine acid suppression. When pharmacological treatment is required, indications should be evaluated strictly: acid-suppressing therapy is warranted only for confirmed peptic ulcer, erosive gastritis, GERD, or high bleeding risk, and should not be used routinely for transient nausea or loss of appetite.

Prefer H₂RAs over proton pump inhibitors (PPIs). H₂RAs inhibit gastric acid more mildly and perturb the gut microbiota less than PPIs, and should be the first choice when acid suppression is needed. If a PPI is required, select agents with weaker CYP2C19 inhibition (pantoprazole, rabeprazole) to minimize pharmacokinetic interactions with antituberculosis drugs (rifampin, isoniazid).

Limit use to the shortest effective course. Cohort evidence shows that PPI use beyond 8 weeks induces significant gut microbiota shifts, with only partial recovery 4–8 weeks after discontinuation. PPI use should therefore be restricted to the shortest effective duration, without long-term maintenance in the absence of indication; in patients receiving standard 6-month ATD regimens, cumulative acid-suppressant exposure should be weighed when assessing treatment risks.

Strengthen individualized assessment in high-risk populations. Caution is warranted in patients at high risk of baseline dysbiosis (diabetes, HIV infection, malnutrition, or recent broad-spectrum antibiotic use). Baseline gut microbiota assessment before starting a PPI and dynamic monitoring during treatment are recommended in such patients to enable proactive risk management.

#### 6.2.2 The potential of microbiota-targeted interventions as adjuvant therapies

The modifiability of the gut microbiota provides a theoretical basis for its use as an adjunctive target in ATD. Currently, microbiota-targeted intervention strategies, represented by probiotics, prebiotics, synbiotics, and fecal microbiota transplantation (FMT), are receiving increasing attention for their application in ATD.

ATB-DILI is the most studied target of probiotic adjunct therapy, acting mainly through the gut–liver axis via inhibition of the LPS/TLR4/NF-*κ*B pathway. *Lactobacillus casei* strain Zhang, the most extensively studied strain (86), alleviates isoniazid- and rifampin-induced liver injury in animals by strengthening the intestinal barrier (upregulating ZO-1 and claudin-1), reshaping the microbiota, reducing serum LPS, and downregulating hepatic NF-*κ*B/MyD88; clinical data confirm that *Lactobacillus casei* reduces cholestasis-related liver enzyme elevations during TB treatment (87). *Bacteroides fragilis* 839 similarly protected against HRZE-induced liver injury in mice (reduced ALT, AST, and MDA; improved histopathology) (88), and a synbiotic containing *Lactobacillus sakei* CVL-001 reduced both Mtb burden and hepatic inflammation (increased regulatory T cells and IL-10, reduced ALT and hepatocyte death) (89). *Bifidobacterium* has also been implicated in adaptive responses to isoniazid-induced liver injury (90).

Clinical evidence on probiotic supplementation for ATD-related gastrointestinal adverse reactions has accumulated. In a randomized, open-label trial of 429 adults during the intensification phase, a proprietary *Lactobacillus casei* preparation reduced the incidence (29.4% and 37.6% vs. 50.0% in controls) and duration of gastrointestinal adverse reactions (91); however, the open-label, non-placebo-controlled, single-center design and reliance on self-reported symptoms limit its reliability and generalizability. A prospective cohort of 177 patients in southern India reported fewer adverse drug reactions with probiotics (14.8% vs. 61.3%, *p* < 0.001) and a lower systemic inflammatory response index, but no significant change in treatment success or quality of life (92); as a non-randomized, single-center study with modest sample size, it is susceptible to selection bias and residual confounding, and its null treatment-success findings caution against overinterpretation. Mechanistically, animal studies suggest that *Lactobacillus casei* prevents ATD-induced intestinal damage by modulating the microbiota and SCFA metabolism (93), and *Lactobacillus sakei* CVL-001 alleviates gastrointestinal and hepatic adverse reactions by suppressing T-cell-mediated inflammation and increasing regulatory T cells and IL-10 (89); a double-blind trial of *Lactobacillus casei* Shirota is ongoing. Importantly, probiotic effects are strain- and preparation-specific, and the evidence base, predominantly small, short-term, single-center studies from China and India, largely confined to the intensification phase (≤8 weeks), remains insufficient to recommend probiotics as routine adjunctive therapy; no trial to date has shown an effect on clinically relevant outcomes such as sputum culture conversion or treatment success, underscoring the need for blinded, placebo-controlled, multi-center trials with standardized strains and long-term follow-up.

FMT, a core strategy for microbiota restoration with proven efficacy in diseases such as *Clostridium difficile* infection, is being explored as an adjunctive treatment for tuberculosis (particularly intestinal tuberculosis) by modulating dysbiosis and immune responses. However, its application in active TB faces challenges of infection control, immune-status assessment, and pathogen transmission, and its safety and efficacy require systematic evaluation (94–96).

### 6.3 Limitations and future directions

#### 6.3.1 Key scientific questions requiring urgent resolution

The most pressing gap is the absence of direct evidence on how combined ATD and acid-suppressing agents affect clinical outcomes. Although the “microbiota–immunity–therapeutic efficacy” cascade is mechanistically plausible, no large prospective cohorts or randomized trials have verified whether the combination alters sputum culture conversion, lesion resolution, or treatment failure rates; current evidence rests largely on animal and *in vitro* studies, leaving a substantial translational gap.

Second, gut microbiota biomarkers that predict treatment response and identify high-risk patients early are a priority for translational research. Candidate indicators discussed earlier (depletion of SCFA-producing bacteria, expansion of oral-derived microorganisms, and altered secondary bile acid levels) could serve as predictive biomarkers. Baseline and on-treatment microbiota sampling, combined with metabolomics and immunophenotyping, should support multidimensional models to identify patients at risk of poor response from combined acid-suppressing therapy.

Third, personalized acid-suppression strategies are needed. Given heterogeneity in baseline microbiota, immune function, and drug metabolism, no uniform regimen can balance the risk–benefit ratio for all patients; a stratified decision-making framework based on microbiota assessment should determine who can safely use PPIs and who should preferentially receive H₂RAs or non-pharmacological management.

#### 6.3.2 Established facts vs. theoretical inferences

The cascade model rests on extensive mechanistic evidence, but established facts must be distinguished from hypothetical inferences. Established: ATDs and PPIs each independently reduce microbial diversity, deplete SCFA-producing genera, and alter metabolite profiles (butyrate, secondary bile acids), and the gut–lung axis operates in animals and humans. Hypothetical and awaiting validation: true synergy between the two drug classes, direct impairment of pulmonary immune surveillance by dysbiosis, and translation into clinically relevant outcomes (sputum culture conversion, lesion resolution, DILI). As supporting data derive mainly from animal and *in vitro* studies without prospective human evidence, the cascade should be viewed as a hypothesis-generating framework; future work should test each link in prospective cohorts or trials.

## 7 Conclusion

In this review, we have synthesized a theoretical framework, the “microbiota–immunity–efficacy” cascade, to conceptualize how the combination of ATDs and acid suppressants might impair tuberculosis treatment outcomes through a sequence of effects: additive or even synergistic microbiota depletion, dual blockade of metabolic pathways, immune homeostasis dysregulation, and potentially worsened clinical outcomes. In its present form, this cascade should be regarded as a hypothesis-generating framework rather than a proven causal chain. It is established that ATDs and PPIs each independently alter gut microbiota composition, SCFA and secondary bile acid metabolism, and gut–lung axis signaling, whereas the downstream links, from dual-exposure dysbiosis to clinically meaningful outcomes such as delayed sputum culture conversion, poor lesion resolution, and drug-induced liver injury, remain hypothetical and lack direct confirmation from prospective human studies.

Based on the mechanistic evidence established to date, we recommend that clinicians strictly adhere to the indications for acid-suppressing agents during ATD, prioritize H₂RAs over PPIs when pharmacological intervention is necessary, and limit treatment to the shortest effective duration. Microbiota-targeted interventions, particularly probiotics such as *Lactobacillus casei*, have shown potential as adjunctive strategies for mitigating adverse reactions in preclinical studies and small, largely single-center clinical trials, though their long-term efficacy requires further validation.

Finally, large-scale prospective cohort studies and randomized controlled trials are urgently needed to test each link of this cascade, to confirm whether co-prescription of acid suppressants with ATDs directly affects clinically relevant outcomes, and to establish microbiota-based biomarkers for identifying high-risk patients and predicting therapeutic response, thereby advancing acid-suppression strategies from empirical use toward evidence-based, precision management. Until such evidence is available, the proposed cascade should serve to guide cautious clinical practice and research prioritization rather than definitive claims of causality.
